# Does serial procalcitonin monitoring predict clinical outcomes in children with sepsis? A diagnostic stewardship study

**DOI:** 10.1017/ash.2025.10032

**Published:** 2025-06-03

**Authors:** Beenish Rubbab, Samuel Davila, Jessica Moreland, Sarah Firmani, Zachary Most

**Affiliations:** 1 Division of Infectious Disease, Department of Pediatrics, University of Texas Southwestern Medical Center, Dallas, TX, USA; 2 Division of Pediatric Critical Care, Department of Pediatrics, University of Texas Southwestern Medical Center, Dallas, TX, USA; 3 Department of Microbiology, University of Texas Southwestern Medical Center, Dallas, TX, USA; 4 Children’s Medical Center, Dallas, TX, USA

## Abstract

**Objective:**

To determine if the initial procalcitonin level and the serial trend of procalcitonin levels in blood were predictive of clinical outcomes in children with sepsis.

**Design:**

A retrospective cohort study

**Setting:**

One primary-to-quaternary care pediatric healthcare system from May 2020 to May 2022

**Participants:**

Encounters for children 1 to 18 years old with a sepsis ICD-10 diagnosis code and clinical sepsis were included.

**Methods:**

Procalcitonin clearance at 48 hours (CL-PCT_48_) was defined as the difference in procalcitonin values drawn on admission and at 48 hours divided by initial procalcitonin value. The primary outcome was early clinical stability. Receiver operating characteristic analysis was performed to measure the correlation of CL-PCT_48_ and initial procalcitonin value (PCT_0_) with the outcomes.

**Results:**

320 unique encounters met the clinical criteria of sepsis with at least two procalcitonin values. 187 encounters had procalcitonin collected at eligible times. The mean age of the participants was 9 years and 8 months, 103 (55%) were male, and 74 (40%) were Hispanic. 78 (41.7%) individuals had good early clinical response, and 177 (94.7%) survived. There was no correlation identified between CL–-PCT_48_ and early clinical stability (area under ROC curve [AUC] = 0.57, 95% CI 0.48–0.65) or mortality (AUC = 0.60, 95% CI 0.43–0.76). There was also no correlation between PCT_0_ and early clinical stability (AUC = 0.47, 95% CI 0.39–0.56) or mortality (AUC = 0.50, 95% CI 0.29–0.72).

**Conclusion:**

Procalcitonin clearance at 48 hours after admission did not predict early clinical stability in children with sepsis.

## Introduction

Sepsis is a life-threatening condition and a common indication for pediatric hospital admission across the globe. Sepsis is a significant public health concern with a case fatality rate of around 25% in children.^1–3^ Various biomarkers have been studied for diagnosing, guiding management decisions, and informing prognosis for patients with sepsis.^4^ Certain biomarkers, including C-reactive protein and procalcitonin, have been the most frequently studied and proposed to have a role in assisting with clinical evaluation for sepsis.^5^

Over the past decade, single procalcitonin values have emerged as a useful tool to reduce antibiotic exposures and for diagnosis of infections in difficult clinical scenarios.^6–8^ However, patients frequently get serial procalcitonin values measures during a hospitalization for sepsis. Prior studies have shown mixed results on the utility of serial procalcitonin monitoring.^9–11^ Additionally, there is a paucity of data for the pediatric population.

There are several potential drawbacks to overuse of clinical biomarker testing, such as increased patient charges, and misleading results that inappropriately lead to changes in therapy. Therefore, if the repeat biomarker tests provide little or no clinical information, serial biomarker testing could be a good target for diagnostic stewardship interventions. We sought to assess the clinical utility of early serial procalcitonin monitoring in children with sepsis.

## Methods

A retrospective cohort diagnostic study was performed at a primary-to-quaternary care pediatric healthcare system in Texas from May 2020 to May 2022. Patients were initially identified by extracting all encounters that had an ICD-10 diagnosis code for sepsis (A41.XX) and at least two procalcitonin values collected during the admission. Extracted demographics, vital signs, and laboratory values, along with manual chart review, were used to determine patient inclusion and exclusion.

Clinical encounters were included if they met all of the following: assigned ICD-10 code for sepsis (A41.XX), the patient met clinical criteria for sepsis (as defined by the International Consensus Conference on Pediatric Sepsis^12^), symptoms for sepsis developed prior to or within 48 hours of admission, and at least two procalcitonin values were collected within 60 hours of admission, with the first procalcitonin value drawn between 0–12 hours after admission and another drawn between 36–60 hours after admission.

Encounters were excluded if the patient had a condition that could affect inflammation biomarkers: a past medical history of autoimmune diseases (including systemic lupus erythematosus, hemophagocytic lymphohistocytosis, scleroderma, systemic vasculitis, inflammatory bowel disease, or juvenile rheumatoid arthritis); the index admission revealed a new diagnosis of malignancy or autoimmune disorder; the index admission was for trauma or drug overuse, or the patient had fungus grown from a sterile site culture without bacterial growth.

The exposure of interest was procalcitonin clearance at 48 hours as a continuous variable. Procalcitonin clearance (CL-PCT_48_) was defined as the difference in procalcitonin values drawn on admission (PCT_0_) and 48 hours after admission (PCT_48_) divided by initial procalcitonin value: CL-PCT_48_ = [(PCT_0_-PCT_48_) x 100%]/PCT_0_. The initial value, PCT_0_, included a PCT value collected at 0 (inclusive) to 12 (exclusive) hours after admission. PCT_48_ included a PCT value collected at 36 (inclusive) to 60 (exclusive) hours. If more than one PCT level was collected during the interval, the value collected closest to hour 0 or hour 48 was used. CL-PCT_48_ can take any value from negative infinity to positive 100%. Negative values indicate the PCT level increased, and greater values indicate a larger decline in PCT.

The primary outcome was a dichotomous variable of early clinical stability at 120 hours, defined as a composite measure of the following four criteria for 24 consecutive hours or more at 120 hours after admission: normal body temperature, no vasopressors, no supplemental oxygen requirement above the baseline for patient, and no renal replacement therapy in addition to the baseline for the patient if applicable. The secondary outcome was defined as all-cause in-hospital mortality.

Procalcitonin values were measured from serum or plasma sample using the Abbott Alinity immunoassay analyzer (Abbott Park, IL). Early clinical stability was determined by manual chart review.

Comparisons of the demographics between those who were included and excluded solely due to timing of procalcitonin levels was done to investigate potential sources of bias. A receiver operating characteristic (ROC) curve was plotted to assess correlation between CL-PCT_48_ and outcomes and between PCT_0_ and outcomes. Area under the ROC curve (AUC) with 95% confidence intervals (CI) were calculated. The null hypothesis was that the ROC AUC = 0.5. For 187 encounters this study was powered at >99% to detect an AUC > 0.7 for the primary outcome assuming 50% of encounters meet early clinical stability. All statistical calculations were completed in Stata v16.1 (StataCorp, College Station, Texas).

Conditions that were likely to be associated with changes in procalcitonin levels were excluded to reduce the likelihood of confounding. We performed subgroup analyses for encounters of patients with bacterial growth from sterile body site or urine culture, a positive blood culture, a PCT_0_ value greater than 1 ng/mL, and with a diagnosis of multisystem inflammatory syndrome in children associated with COVID-19 (MIS-C).

The study was reviewed by the University of Texas Southwestern Institutional Review Board and was determined to be exempt from review due to minimal or no risk to subjects.

## Results

**Figure 1.**
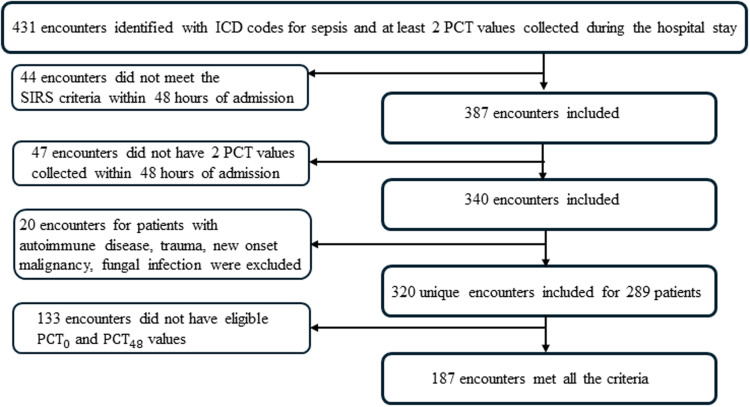
Study design flow chart.

There were 431 encounters identified with an ICD-10 code for sepsis and at least two procalcitonin values collected. 320 of these encounters for 289 unique patients met sepsis criteria, had at least one PCT collected in the first 48 hours, and did not have an autoimmune condition or fungal infection (Figure 1). The median number of PCT levels was 4 (range 2 – 111, IQR 3–7), but 15% of encounters had 10 or more PCT values collected (Supplemental Figure 1). There were 187 encounters that had PCT measurements done at eligible times to be included in the primary analysis (Figure 1).

The median age of patients was 9 years, and 55% were male. A plurality of patients were identified as Hispanic, with non-Hispanic White and non-Hispanic Black patients equally represented. In 69% of encounters, patients were directly admitted to intensive care unit. There were no significant differences in demographics between the 187 encounters included in the analysis and the 133 that were excluded due to procalcitonin timing. Out of 187 included encounters, 57 had a positive sterile body site or urine culture, of which 30 had positive blood cultures (Table 1, Supplemental Table 1). 42% of encounters met the early clinical stability, and 95% survived.

**Table 1.** Demographic and clinical characteristics of the patients with unique encounters included in the study

Variables	CL-PCT_48_ eligible encounters N = 187	CL-PCT_48_ non-eligible encounters N = 133
Age, mean ± SD (years)	9.5 ± 4.9	9.8 ± 4.9
**Gender,** ***n*** **(%)**		
Male	103 (55%)	71 (53%)
Female	84 (45%)	62 (47%)
**Race and Ethnicity,** ***n*** **(%)**		
Hispanic	74 (40%)	57 (43%)
Non-Hispanic Asian	7 (4%)	9 (7%)
Non-Hispanic Black	48 (26%)	22(17%)
Non-Hispanic White	49 (26%)	32 (24%)
Non-Hispanic Others	9 (4%)	13 (10%)
Financial class, *n* (%)		
Private	50 (27%)	44 (33%)
Public	134 (72%)	87 (65%)
Self-pay/ Others	3 (2%)	2 (2%)
**Admitting Unit/Service,** ***n*** **(%)**		
Critical Care	129 (69%)	83 (62%)
General Pediatric or subspeciality	58 (31%)	50 (38%)
Positive culture from sterile body site or urine*, *n* (%)	57 (30%)	39 (30%)
Positive blood culture, *n* (%)	30 (16%)	15 (11%)
Empiric antibiotic targeted the identified pathogen, *n* (%)	43 (77%)	33 (85%)
Comorbidities, *n* (%)	75 (40%)	46 (35%)
Asthma	9 (5%)	6 (4.5%)
Chronic Lung Disease	54 (29%)	41 (31%)
On home oxygen	42 (22%)	33 (25%)
Not on home oxygen	12 (6%)	8 (6%)
Immunocompromised host	18 (9.6%)	17 (13%)
Primary immunodeficiency	1 (0.5%)	2 (1.5%)
Leukemia/Lymphoma	5 (2.7%)	10 (7.5)
Solid organ tumors	2 (1%)	4 (3%)
Solid organ transplant	8 (4.3%)	1 (0.8)
Bone marrow/ stem cell transplant	0	0
Hemophagocytic lymphocytosis	1 (0.5%)	0
Central line dependence	9 (5%)	7 (5%)
Cerebral Palsy/ encephalopathy	46 (24%)	41 (31%)
Renal disease **	26 (14%)	25 (19%)
Known genetic mutation	17 (9%)	11 (8%)
Congenital heart disease	15 (8%)	8 (6%)
Others***	19 (10%)	9 (8%)

* Included blood, tissue, body fluid, wound, abscess, and urine.

** Chronic kidney disease, end-stage renal disease, neurogenic bladder, urinary retention, vesicoureteral reflux, requiring intermittent or continuous catheter, and anatomically abnormal kidney.

*** Intrauterine growth retardation, failure to thrive, presence of ventricular peritoneal shunt, hematological diseases.

**Figure 2.**
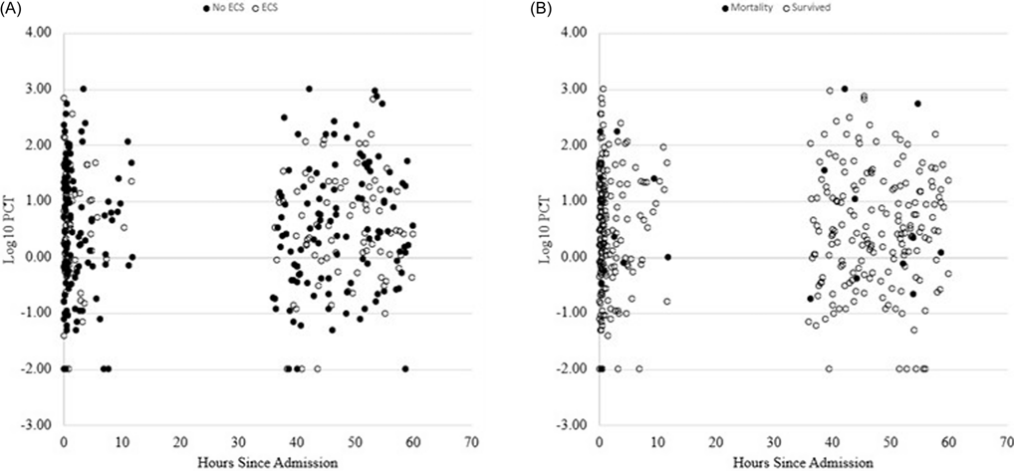
Scatter plots; A) PCT clearance in 48 hours and PCT_0_ in encounters with early clinical stability and without early clinical stability. B) PCT clearance in 48 hours and PCT_0_ in encounters for survivors and non survivors.

**Figure 3.**
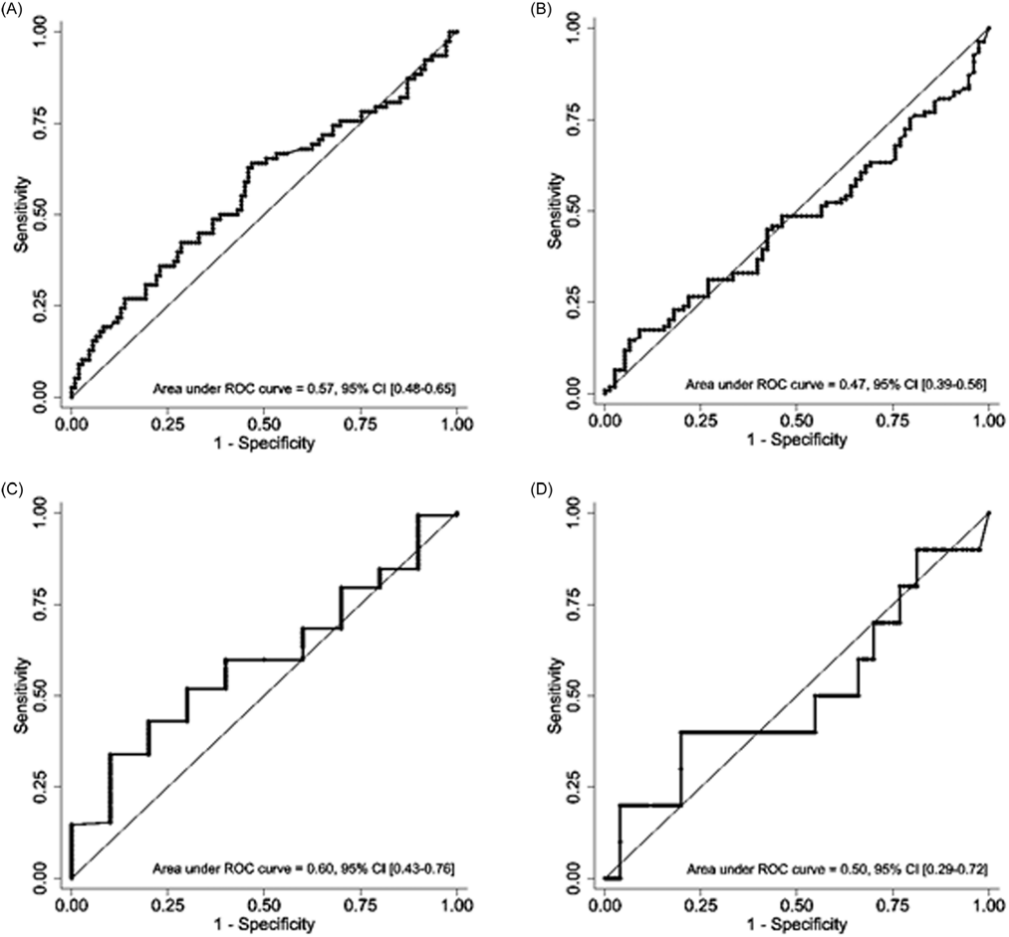
Receiver operating curves for PCT_0_ and PCT_48_ eligible encounters: A) PCT clearance in 48 hours and early clinical stability. B) PCT_0_ and early clinical stability. C) PCT clearance in 48 hours and all-cause mortality. D) PCT_0_ and all-cause mortality.

In the 187 encounters that met eligibility criteria, the ROC-AUCs for early clinical stability were 0.57 (95% CI 0.48–0.65) and 0.47 (95% CI 0.39–0.56) for CL-PCT_48_ and PCT_0_, respectively. For all cause hospital mortality, AUCs were 0.60 (95% CI 0.43–0.76) and 0.50 (95% CI 0.29–0.72) for CL-PCT_48_ and PCT_0,_ respectively (Figures 2 and 3). Since the results were not statistically significant, we did not seek to identify an optimal cutoff value for CL-PCT_48_.

**Figure 4.**
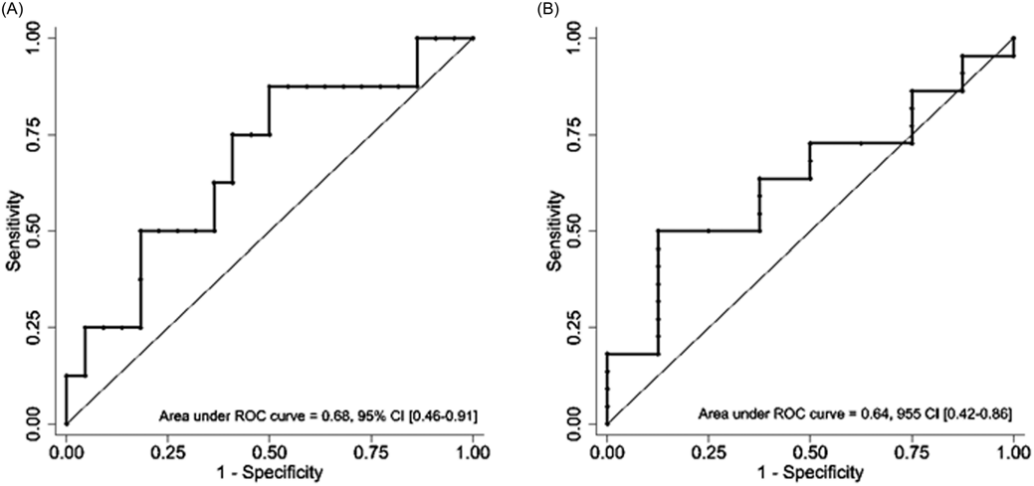
Receiver operating curve for subgroup of encounters with positive blood cultures: A) PCT clearance in 48 hours and early clinical stability. B) PCT_0_ and early clinical stability.

In the analysis of a subgroup of encounters with positive blood cultures (n = 30), the ROC-AUCs for early clinical stability (n = 8) were 0.68 (95% CI 0.46–0.91) and 0.64 (95% CI 0.42–0.86) for CL-PCT_48_ and PCT_0_, respectively. There were two deaths in this subgroup but both deaths occurred in individuals with PCT_0_ above or equal to 174.15 ng mL^−1^. Due to the small number of events, we did not generate ROC curves for all-cause mortality in this subgroup (Figure 4).

In the analysis of a subgroup of encounters with initial procalcitonin value of >1 ng/ml, the ROC-AUCs for early clinical stability were 0.57 (95% CI 0.47–0.68) and 0.55 (95% CI 0.44–0.65) for CL-PCT_48_ and PCT_0_, respectively. For all-cause hospital mortality, the AUCs were 0.72 (95% CI 0.54–0.89) and 0.69 (95% CI 0.41–0.97) for CL-PCT_48_ and PCT_0_, respectively (Supplemental Figure 2). The optimal cutoff of CL-PCT_48_ for predicting all-cause mortality was ≥ −336%, which had a sensitivity of 75% and a specificity of 50%.

In the subgroup of encounters with diagnosis of MIS-C, the ROC-AUCs for early clinical stability were 0.68 (95% CI 0.46–0.91) and 0.64 (95% CI 0.42–0.86) for CL-PCT_48_ and PCT_0_, respectively. There were no deaths in the MIS-C subgroup (Supplemental Figure 3).

## Discussion

In our study, neither initial nor early serial procalcitonin values were predictive of early clinical stability in pediatric patients with sepsis. Similar results were observed in subgroup analyses for patients with laboratory evidence of bacterial infection, although those with documented bacterial bloodstream infections had a trend towards higher a ROC-AUC. For patients with initial procalcitonin values that were greater than 1 ng/mL there was a statistically significant correlation between procalcitonin clearance at 48 hours and all-cause in-hospital mortality, but there were few deaths overall. The subgroup of patients with a diagnosis of MIS-C were also noted to have abnormal initial procalcitonin levels and showed decreasing procalcitonin over the hospital course but were not statistically significant to predict the early clinical stability. These results suggest that frequently rechecking procalcitonin levels in children with sepsis has little clinical utility except a possible small predictive value in subgroups with high initial procalcitonin levels and those with confirmed bacterial bloodstream infections.

Our results have some important differences and similarities to previously published reports regarding procalcitonin clearance. In a prospective study in a group of critically ill adult patients with septic shock, decrease in PCT levels on day 5 greater than 7.8 ng mL^−1^ was considered as the cut off in predicting survival with sensitivity of 35% and specificity of 85%.^13^

A recent metanalysis supported that procalcitonin non-clearance predicts mortality in adults with severe sepsis but the differences in inclusion criteria for sepsis may account for some heterogeneity.^14^ A cross-sectional study in pediatric population with lymphoreticular malignancies admitted with fever and neutropenia suggested that serial procalcitonin monitoring can be useful in predicting adverse outcomes, defined by prolonged fevers, development of sepsis, persistence of severe neutropenia, positive microbiological culture, organ dysfunction, or death.^15^ Our study included a more heterogenous patient population and a different outcome measure, which may reflect why we did not observe similar results. A prospective study used normalization of procalcitonin for neonatal sepsis to decide optimal duration of antibiotics and concluded that there was no increase in morbidity and mortality at 4-week follow-up.^16^

Our study has several strengths. The population included was a pragmatic set of hospitalized children with clinical diagnoses of sepsis who had multiple procalcitonin values. This realistically reflects a population that are undergoing serial inflammatory marker measurements for “trending”, rather than prior studies that focused on populations only with confirmed infections, severe sepsis, or risk factors like neutropenia. This highlights that inflammatory marker “trending” is a good target for diagnostic stewardship efforts. Our data set also highlights that PCT might be a nonspecific marker of inflammation, observing the high values in patients with MIS-C. Since mortality is rare in hospitalized children with sepsis in the USA, we used an outcome of early clinical stability, which is more closely tied to the response to the initial interventions for the patient and had enough negative outcomes to power this study.

Our study is limited due to the small sample size. This study simplifies the patient’s situation to two procalcitonin values and does not account for all the intricacies of patient care, so we cannot conclude that trending PCT is not useful in all situations. We did not compare the PCT values with the patient-specific objective data, including vital signs, needs for pressors, physical examination, and other markers of infections like white blood cell count and C reactive protein, which could further compare if PCT has additional value above these other measures.

Randomized control trials evaluating patient outcome based on standardized PCT monitoring strategies are needed to observe the utility of repeating serial procalcitonin levels. Serial procalcitonin monitoring may provide misleading results that affect clinical management of the patients, which may lead to inappropriate antimicrobial use, prolonged hospitalization, and over utilization of available resources.

## Supporting information

Rubbab et al. supplementary material Rubbab et al. supplementary material

## Supplementary material

The supplementary material for this article can be found at https://doi.org/10.1017/ash.2025.10032
