# Supplementary material for: Does serial procalcitonin monitoring predict clinical outcomes in children with sepsis? A diagnostic stewardship study
Source: Antimicrob Steward Healthc Epidemiol. 2025 Jun 3;5(1):e124. doi: 10.1017/ash.2025.10032 (PMC12171929; doi:10.1017/ash.2025.10032)
Supplement: Rubbab et al. supplementary material [file S2732494X25100326sup001.docx]

**Supplementary Data:**

PCT level below 0.5µg/L is considered normal for Abbot Alinity immunoassay analyzer. A few of PCT values were reported as <0.04, >50, and >1000 ng mL^-1^. To convert the data for numerical analysis we chose 0.01 for the PCT values < 0.04, 70 (between 50-95^th^ percentile-6 patients) for PCT values >50, 1000 for PCT >1000 ng mL^-1^.

Supplementary table 1: Additional characteristics of the cohort

| Variables  N (%) | PCT_48_ eligible cohort  N=187 | PCT _48_ non eligible cohort  N= 133 |
| --- | --- | --- |
| Years of encounters  2020  2021  2022 | 48 (36%)  64 (48%)  21 (16%) | 52 (28%)  88 (47%)  47 (25%) |
| Hospital campuses  DALLAS  PLANO | 108 (81%)  25 (18%) | 157 (84%)  30 (16%) |
| ICD- 10 Codes  A41.01  A41.02  A41.1  A41.2  A41.3  A41.51  A41.52  A41.53  A41.59  A41.81  A41.89  A41.9 | 16 (9%)  7 (4%)  2 (1%)  1 (0.5%)  1 (0.5%)  7 (4%)  1 (0.5%)  1 (0.5%)  3 (2%)  4 (2%)  45 (24%)  99 (53%) | 3 (2%)  2 (2%)  0  0  0  14 (11%)  5 (4%)  0  5 (4%)  1 (2%)  30 (22%)  73 (55%) |
| Financial status  Private  Public  Others/self-pay | 50 (27%)  134 (72%)  3 (2%) | 44 (33%)  87 (65%)  2 (2%) |
| Organisms isolated from sterile body site or urine cultures  Gram positive  Gram negative | 57 (30%)  33 (58%)  28 (49%)  *5 patients had polymicrobial infection | 39 (30%)  11 (28%)  29 (74%) |
| Empiric antibiotic started in patients with positive culture data  Optimal antibiotic coverage | 57 (100%)  43 (75%) | 39 (100%)  33 (85%) |
| Pathogens isolated from entire cohort  MSSA MRSA  Coagulase negative Staphylococcus Enterococcus sp  Streptococcus pneumoniae  Group A Streptococcus  Enterobacter sp  Ecoli  Pseudomonas aeruginosa  Klebsiella sp  Proteus sp  Cronobacterium sp  Salmonella sp  Serratia sp  Morganella sp  Abiotrophia sp  Aggrebacterium sp  Bacteroides fragilis  Hemophilus influenza  Moraxella catarrhalis  Stenotrophomonas sp  Mycobacterium sp | N=187  15 (8%)  6 (3%)  3 (2%)  8 (4%)  1 (0.5%)  1 (0.5%)  1 (0.5%)  15 (8%)  1 (0.5%)  5 (3%)  0  1 (0.5%)  1 (0.5%)  1 (0.5%)  1 (0.5%)  1 (0.5%)  0  1 (0.5%)  1 (0.5%)  0  1 (0.5%)  1 (0.5%) | N=133  4 (3%)  2 (2%)  1 (1%)  3 (2%)  0  1 (0.75%)  0  19 (14%)  3 (2%)  3 (2%)  3 (2%)  0  0  0  0  0  1 (0.75%)  0  0  1 (0.75%)  0  0 |

| Supplemental Table 2. Components of composite outcome measure (N = 187 individuals) | | | | |
| --- | --- | --- | --- | --- |
|  | Any time prior to 120 hours after admission | | At 120 hours after admission | |
| Outcome | n | % | n | % |
| Composite - No early clinical stability | 187 | 100% | 109 | 58.3% |
| Fever or hypothermia | 187 | 100% | 44 | 23.5% |
| Vasopressors | 85 | 45.5% | 14 | 7.5% |
| Supplemental oxygen | 183 | 97.9% | 91 | 48.7% |
| Renal replacement therapy | 11 | 6.1% | 11 | 6.1% |

The composite outcome of early clinical stability was met if the individual did not meet any of the four criteria for 24 or more consecutive hours at 120 hours after admission. No early clinical stability means at least one of the four criteria were met at 120 hours.
The denominator for the renal replacement therapy is 180, which excludes 7 individuals who were on dialysis at baseline. The renal replacement therapy criteria was not used in the composite measure for patients on dialysis at baseline.


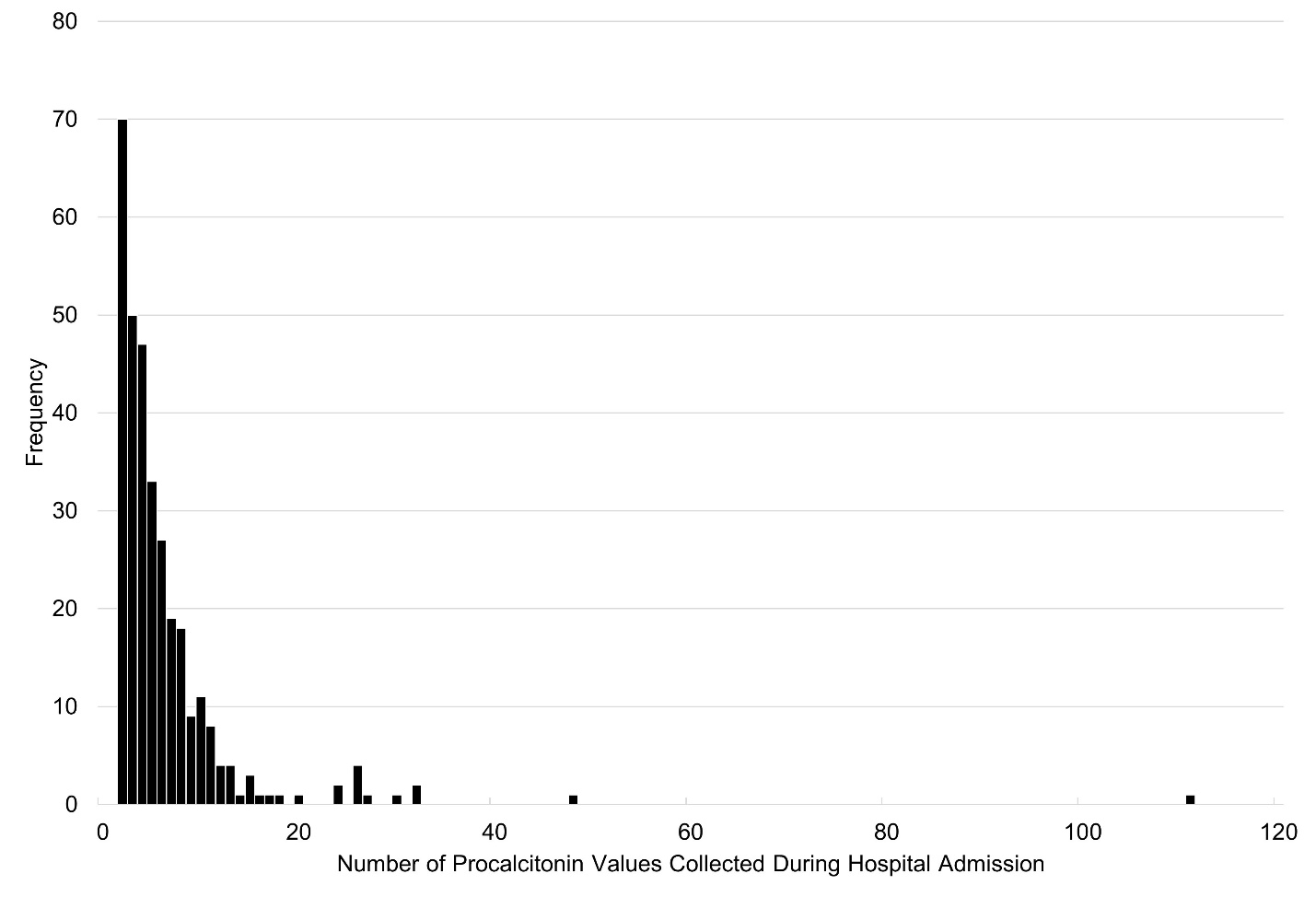


Supplementary Figure 1: Histogram showing frequency of encounters with the respective number of procalcitonin values collected for 320 encounters.

A) B)


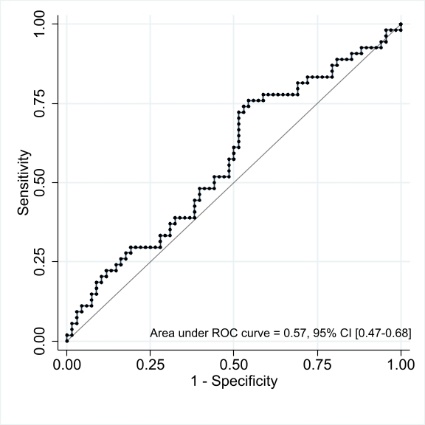

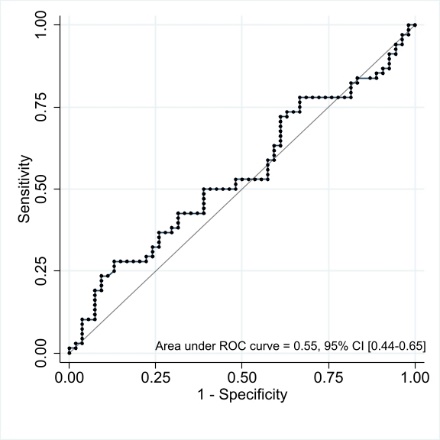


C) D)


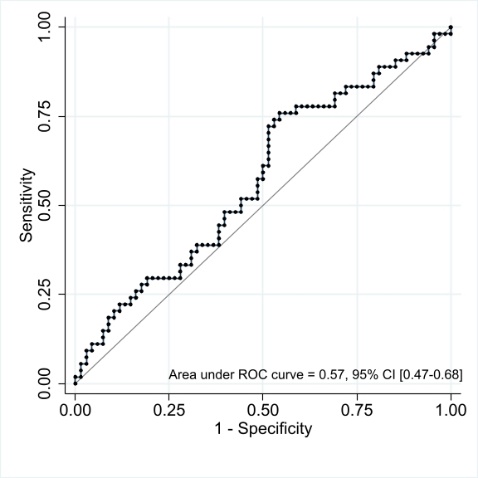

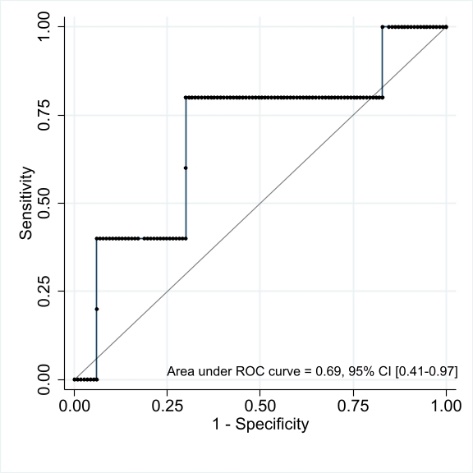


Supplementary figure 2: Receiver operating characteristic curve for subgroup of encounters with with PCT0 >1 (n=122, out of 122, 54 achieved early clinical stability) for initial PCT value (PCT_0_) and PCT clearance in 48 hours for primary and secondary outcome A) PCT clearance in 48 hours and early clinical stability. B) PCT_0_ and early clinical stability. C) PCT clearance in 48 hours and all-cause mortality. D) PCT_0_ and all-cause mortality.

A) B)


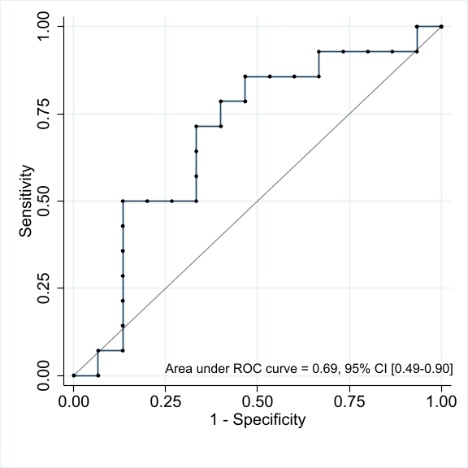

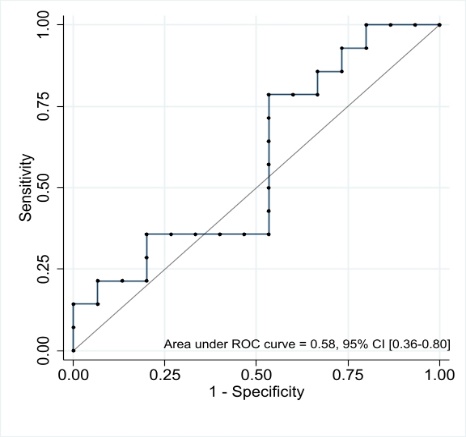


Supplementary figure 3: Receiver operating characteristic curve for subgroup of encounters with diagnosis of MIS-C (n=29, out of 29, 15 achieved clinical stability) for initial PCT value (PCT_0_) and PCT clearance in 48 hours for primary and secondary outcome A) PCT clearance in 48 hours and early clinical stability. B) PCT_0_ and early clinical stability. No deaths in this group
